# Mass Drug Administration and Worms Experience in Africa: Envisage Repurposing Ivermectin for SARS-COV-2

**DOI:** 10.4269/ajtmh.20-0295

**Published:** 2020-05-26

**Authors:** Claire Njeri Wamae

**Affiliations:** 1 School of Pharmacy and Health Sciences, United States International University-Africa, Nairobi, Kenya;; 2 Kenya Medical Research Institute, Nairobi, Kenya

I have spent over 40 years studying parasitic worms and the drugs we use to treat them. With this background, I suggest that large-scale annual mass drug administration for preventive chemotherapy of neglected tropical diseases may be contributing to keeping COVID-19 cases in check and below projections in Africa.

My fascination with worms started in 1979, while undertaking a food science major in college. I was dismayed to learn of tiny blackflies that breed in fast-flowing rivers in Africa and transmit river blindness! What horrified me most about the strange disease was the textbook picture depicting a naked blind African adult man, led on a cane by a little boy (naked also). I thought, propaganda:

This couldn’t be true, it isn’t fair, I come from Africa and people don’t walk around naked! Besides, growing up in Kenya, I had never seen or heard of anyone blinded by the bite of a fly….

I reasoned that it would make more sense to study worms and prioritize restoration of health, as availing quality foods to wormy communities would be a disservice. This shocking experience was my turning point from studying food science to studying parasitology.

I have often been reminded of river blindness throughout my professional career, whether working in Africa, attending meetings at the Headquarters of the World Bank in Washington or the WHO in Geneva, or teaching in classrooms the world over. The two river blindness monuments in Washington and Geneva, unlike my earlier entomology textbook picture in Ohio, are inspiring. I believe both monuments express pride in progressively controlling river blindness disease, millions of averted river blindness, and hectares of fertile land reclaimed for agriculture through concerted global efforts.

River blindness, also called onchocerciasis, is one of the five among 20 neglected tropical diseases, amenable to preventive chemotherapy. The other four are lymphatic filariasis (elephantiasis), schistosomiasis (bilharziasis), intestinal helminthiasis (hookworm, whipworm, and roundworm), and blinding trachoma. From the onset, generous drug donations have been provided for several years by pharmaceutical companies: Merck & Co., Inc., Kenilworth, NJ (ivermectin), Eisai Co. Ltd., Japan (diethylcarbamazine), GlaxoSmithKline, United Kingdom (albendazole), Johnson & Johnson, New Brunswick, NJ (mebendazole), Pfizer, United Kingdom (Zithromax), among others. By 2018, up to 1.752 billion treatments were distributed to affected populations throughout endemic areas in the world. As of 2015, 99% of persons infected by *Onchocerca volvulus* globally were living in 31 African countries (http://www.who.int/gho); thus, Africa received the bulk of ivermectin donations.

A casual look at the *O. volvulus* (http://www.who.int/gho) and COVID-19 (http://africacdc.org) maps lacks the typical neglected tropical disease overlaps. In equatorial Africa, we commonly find high prevalence and overlaps of the aforementioned five diseases in same geographical areas (populations). Conversely, COVID-19 cases deviate from this, being higher in the northern and southern temperate areas. To date, the number of confirmed COVID-19 cases in Africa appears to be lower than earlier anticipated. In Kenya, the first imported COVID-19 case was reported on March 13, 2020. According to the Ministry of Health Daily Briefings of April 29, 2020, the number of confirmed cases stood at 384, far less than the country’s projection of 5,000 within 1 month after the index case. Throughout equatorial Africa, the reported confirmed cases fall short of earlier predictions. The continent has reported COVID-19 recoveries and relatively few deaths despite its weak health systems. The observed deviation from earlier projections has spurred various propositions including relatively limited global travel, late introduction of imported COVID-19 cases, limited testing level, high coverage of mandatory tuberculosis vaccination, leading region in mobile money services, and climate. Although it is premature to make any firm conclusions, as it is unclear how the COVID-19 infections will spread on the continent in the coming weeks, I suggest that the impact of preventive chemotherapy of neglected tropical diseases on SARS-COV-2 and, in particular, ivermectin may be reducing susceptibility to COVID-19 across equatorial Africa.

I am unsure of when discussions on ivermectin use in human health started, but I recall hearing senior colleague scientists in passionate conversations on onchocerciasis treatment with ivermectin during two annual O-Now Symposia in Leiden, the Netherlands, in the late 80s. During the second symposium, I presented my first international paper on recovery of new filarial parasites of nonhuman primates in Kenya. Before this, I was at Tulane University and its National Primate Research Center, training on transmission of filariasis in monkeys and, subsequently, working at the Institute of Primate Research, Kenya, in collaboration with the Kenya Medical Research Institute, funded by the WHO. Around that time, there was a focused search for effective, safe, and easily administered treatments for filarial parasites. In Kenya, we wanted to establish *Wuchereria bancrofti* in nonhuman primates to pave way for clinical trials. While screening quarantined, wild-captured, nonhuman primates, we recovered new filarial parasites, *Cercopithifilaria verveti* and *Cercopithifilaria narokensis*. In respect to drug trials for onchocerciasis, a naturally occurring filaria with skin-dwelling microfilariae in nonhuman primates was exciting to the scientific community. Quite soon after the O-Now Symposia, ivermectin was adopted for human use against onchocerciasis. Despite our recoveries and descriptions of new filariae, the laboratory transmission studies failed to establish *W. bancrofti* infection in monkeys, and the studies were terminated.

Ivermectin, derived from avermectin, was discovered in 1975 by Satoshi Omura, Kitasato University, and William C. Campbell, Merck Institute. It is used for treating parasitic worms, mites, and lice in veterinary medicine. On adoption for human use, Merck pharmaceutical committed “to donate ivermectin free of charge for as long as is needed.” It is on the WHO’s list of essential medicines and is the recommended preventive chemotherapy for *O. volvulus*. Subsequent clinical studies in humans established its effectiveness against several parasites, thus increasing the demand for ivermectin donations.

After terminating the laboratory transmission studies, we shifted to treatment of lymphatic filariasis. The recommended treatment for lymphatic filariasis at that time was 6 mg diethylcarbamazine/kg body weight for 12 consecutive days. To fulfill our ethical obligations to the volunteers, who provided blood for harvesting *W. bancrofti* microfilariae in the terminated studies, and explore alternatives to this cumbersome regimen, we enrolled the participants for an ivermectin clinical trial on *W. bancrofti*. On randomization and treatment, xenodiagnoses with clean laboratory-bred mosquitoes were conducted to monitor drug effectiveness in reduction of circulating microfilariae. An exciting study by design ended with much disappointment, as most mosquitoes died almost immediately after feeding. The most embarrassing moment would come when I presented our findings at a departmental seminar! A visiting scientist (American) interjected:

What did you expect? You feed mosquitoes on ivermectin and still expect to get any xenodiagnoses results?

It was not clear why any colleague scientist would opt to abrasively dismiss our findings instead of providing constructive scientific criticism. Anyways, the “negative results” spiced with those condescending remarks effectively ended that drug trial. In malaria research, there have been interesting investigations on the use of ivermectin for control of mosquitoes and as an antimalarial, which gives me much remorse for failure to publish our “negative” results promptly.

I would work with ivermectin in 1992 in Haiti while working as a Guest Researcher at the CDC in Atlanta, Georgia and again in 2005 in a multisite study in Africa and Asia. The objective of the multisite study was to compare treatment coverage by the health system with community-directed distribution. Findings of this study informed rolling out of global mass drug administration for preventive chemotherapy. I have since been on numerous WHO’s advisory worm assignments across Africa and elsewhere.

The swiftness of research for potential vaccines and medications for COVID-19 is unprecedented, with 23 validated therapeutic targets and 536 registered clinical trials by late March 2020. Despite preliminary demonstration of ivermectin effectiveness on SARS-COV-2 in a study in Australia, and concerted Research and Development global efforts, none of the candidate products has been approved so far. Looking toward likely COVID-19 treatment in areas under mass drug administrations, we should anticipate and pay attention to interactions of any drug on clinical trial, or approved treatment for COVID-19 with current neglected tropical diseases chemotherapies, particularly ivermectin. As COVID-19 treatments are rolled out, we should monitor for potential interactions or collateral benefits from the existing, and well-experienced mass drug distribution and supply logistics. The mass drug administration experience and infrastructure may be useful in enhancing access to COVID-19 treatment in Africa. Furthermore, the increased handwashing driven by COVID-19 guidelines and long-term public health interventions to make water and sanitation more accessible may decrease some water- and sanitation-related neglected tropical diseases as well. These propositions should form interesting collaborative, cross-cutting operational research studies that could accelerate achievement of neglected tropical disease road map milestones, enhance our understanding of emerging/reemerging pathogens, and better pandemic preparedness. I believe combining our long experience in neglected tropical disease with the COVID-19 circumstances can create solutions that strengthen health systems and better serve our populations beyond COVID-19 pandemic.

